# Functional genes to assess nitrogen cycling and aromatic hydrocarbon degradation: primers and processing matter

**DOI:** 10.3389/fmicb.2013.00279

**Published:** 2013-09-17

**Authors:** C. Ryan Penton, Timothy A. Johnson, John F. Quensen, Shoko Iwai, James R. Cole, James M. Tiedje

**Affiliations:** ^1^Department of Plant, Soil and Microbial Sciences, Center for Microbial Ecology, Michigan State UniversityEast Lansing, MI, USA; ^2^Division of Gastroenterology, Department of Medicine, University of California San FranciscoSan Francisco, CA, USA

**Keywords:** functional genes, nifH, aromatic hydrocarbon, nirS, primer specificity, clustering analysis, nirK, nitrogen cycling

## Abstract

Targeting sequencing to genes involved in key environmental processes, i.e., ecofunctional genes, provides an opportunity to sample nature's gene guilds to greater depth and help link community structure to process-level outcomes. Vastly different approaches have been implemented for sequence processing and, ultimately, for taxonomic placement of these gene reads. The overall quality of next generation sequence analysis of functional genes is dependent on multiple steps and assumptions of unknown diversity. To illustrate current issues surrounding amplicon read processing we provide examples for three ecofunctional gene groups. A combination of *in silico*, environmental and cultured strain sequences was used to test new primers targeting the dioxin and dibenzofuran degrading genes *dxnA1*, *dbfA1*, and *carAa*. The majority of obtained environmental sequences were classified into novel sequence clusters, illustrating the discovery value of the approach. For the nitrite reductase step in denitrification, the well-known *nirK* primers exhibited deficiencies in reference database coverage, illustrating the need to refine primer-binding sites and/or to design multiple primers, while *nirS* primers exhibited bias against five phyla. Amino acid-based OTU clustering of these two N-cycle genes from soil samples yielded only 114 unique *nirK* and 45 unique *nirS* genus-level groupings, likely a reflection of constricted primer coverage. Finally, supervised and non-supervised OTU analysis methods were compared using the *nifH* gene of nitrogen fixation, with generally similar outcomes, but the clustering (non-supervised) method yielded higher diversity estimates and stronger site-based differences. High throughput amplicon sequencing can provide inexpensive and rapid access to nature's related sequences by circumventing the culturing barrier, but each unique gene requires individual considerations in terms of primer design and sequence processing and classification.

## Introduction

Microbial community composition is most frequently assessed using the 16S rRNA gene marker, either in direct-targeted amplification or seed-based retrieval from metagenomic datasets. However, the linkages between phylogeny and a particular biological function is weak at best. The targeted sequencing of functional genes provides information that directly codes for function and hence provides a functional framework for classification, and by inference, its host's taxonomic identity, at a depth that is not currently attainable with metagenomic libraries. Varying strategies for processing these data have been employed, mainly based on nucleotide sequences. In many cases processed nucleotide sequences are subjected to BLASTn analyses against the non-redundant NCBI database with or without prior clustering. Translated protein sequences have the advantage of more accurately reflecting biological function and thus dissimilarity cutoffs can be more informative than those based on DNA.

Reference database composition significantly influences overall diversity estimates. This is complicated by the inclusion of non-curated sequences whose true biological function is highly uncertain. Bioinformatic approaches, such as Hidden Markov Modeling (HMM) of functional genes based on seed sequences, can be used to mine larger databases for relevant implied gene function. However, there remains uncertainty as to the appropriate filtering cutoffs, as this varies widely among functional genes. Additionally, many issues are implicitly related to initial primer design, which affect both coverage and specificity (Iwai et al., 2011a).

Downstream sequence processing also poses a challenge in the analysis of both community composition and overall diversity. Particularly, the choice of cluster dissimilarity cutoff values and the use of DNA or amino acid sequences as the basis for analysis are especially variable in the literature (Heylen et al., 2006; Palmer and Horn, 2012). Translated sequences better reflect function through residue conservation at key enzymatic sites, and thus protein-based clustering would be expected to better indicate functional relatedness. Lastly, evidence of horizontal gene transfer of these genes and the resulting impact on diversity indices is still largely unknown (Hirsch et al., 1995; Boucher et al., 2003; Heylen et al., 2006; Jones et al., 2008) and warrants attention as more sequences become publicly available.

Different analysis steps are illustrated below for four different ecofunctional genes, i.e., genes that directly code for a protein catalyzing an important ecological process. These steps are particularly important since a common “pipeline” does not exist and would probably not be appropriate for all ecofunctional genes. The output of any pipeline is improved through careful attention at each stage, taking database coverage and unknown genetic diversity into account. For example, the reference databases for the three ecofuncional groups are of different sizes (dioxin-lke aromatic hydrocarbon degradation < denitrification < nitrogen fixation). For the first gene family example, new primers that target the ether-linked, aromatic hydrocarbon degrading genes *dxnA1*, *carAa*, and *dbfA1*, are evaluated to determine primer specificity using a combination of *in silico*, environmental and cultured strain sequences. Next, primer coverage and downstream processing, particularly in regard to choice of clustering dissimilarity cutoffs, are illustrated for the nitrite reductases in denitrification. Lastly, the N-fixing gene *nifH* is used as the example for analyzing differences obtained using both supervised and non-supervised OTU generation.

## Aromatic hydrocarbon-degrading *dxnA*, *dbfA1*, and *carAa* genes

Aromatic hydrocarbons comprise a chemically diverse class of organic compounds that include monoaromatics (e.g., benzene, benzoate), bicyclic (biphenyls,) polycyclic aromatics (e.g., phenanthrene), N-containing heterocyclics (e.g., carbazole) and oxygen-linked polyaromatics (e.g., dibenzofuran, and dibenzo-p-dioxin). Of these, certain chlorinated dioxins are the most problematic due to their persistence and carcinogenicity, requiring the remediation of contaminated soils and sediments. Potential chemical remediation schemes to detoxify dioxin contaminated soils are costly (Kulkarni et al., 2008). Microbial degradation of dioxins has been studied as an alternative method, but evidence of biodegradation is very limited, plus there are few isolated microbes capable of their degradation. Gene-targeted amplicon sequencing is an alternative to culturing isolates to identify novel catalytic biodiversity, as was done with biphenyl dioxygenase (Iwai et al., 2010). Hence, we used gene-targeted amplification and sequencing to characterize dioxygenases with activity toward problematic dioxin chemicals. Three dioxygenases that attack the angular ether linkages in these molecules are known to catalyze the first step of the dioxin degradation pathway: dioxin dioxygenase (*dxnA1*), dibenzofuran dioxygenase (*dbfA1* or *dfdA1*), and carbazole dioxygenase (*carAa*) (Field and Sierra-Alvarez, 2008). A number of PCR primers are reported that can be used to probe samples for aromatic ring-hydroxylating dioxygenase genes (ARDHs) (Iwai et al., 2011b), including a quantitative PCR primer specific to the *dxnA1* in *Sphingomonas wittichii* str. RW1, which is the only well-characterized dioxin degrader (Hartmann et al., 2012). However, no previously published primer set meeting the requirements for amplicon sequencing solely targets dioxygenases active toward dioxins (Iwai et al., 2011b).

### Reference sequence database generation

In order to identify reliable reference sequences with known activity toward dioxins, dioxygenase sequences were obtained through a manual search of the GenBank database (keywords included angular, dioxin, dibenzofuran carbazole and dioxygenase). Amino acid similarity of the harvested sequences (Table A1) showed three groups within the superfamily of Rieske dioxygenases: Group 1, dioxin 1,10a dioxygenase (*dxnA1*) and dibenzofuran 4,4a dioxygenase (*dfdA1*); group 2, dibenzofuran 4,4a dioxygenase (*dbfA1*); group 3, carbazole 1,9a dioxygenase (*carAa*). To harvest additional related sequences, the DDBJ/EMBL/GenBank non-redundant protein database was searched using Hidden–Markov Models built from all reference sequences from each group with HMMER (Eddy, 2009). These results were obtained from the December 2010 release of FunGene (http://fungene.cme.msu.edu, see also Fish et al., 2013). An HMM bits saved score cutoff of 700 was used, and no additional sequences were obtained through this search, which reinforces the low number of sequenced (or cultured) strains with activity toward dibenzo-*p*-dioxin. Specific degenerate primers were designed from amino acid consensus regions (Table A2) and specificity was determined experimentally by PCR using all indicated strain DNA as template with all three primer sets. The primers were specific to only the gene cluster for which they were designed and did not produce amplicons from closely neighboring gene clusters except in the case of the dbfA1 primer set which produced a minor amplification product when *Rhodococcus* sp. RHA1 was the template DNA (Table A1).

### Amplification and sequence processing

Two environmental samples were chosen as template DNA to be used in gene-targeted metagenomics: a well characterized polychlorinated biphenyl (PCB)-contaminated rhizosphere soil (Leigh et al., 2007), and a pristine Kansas prairie soil (KS) (Williams et al., 2011), from the Konza Prairie (39°05′N, 96°35′W). Both of these soils should contain polyaromatic hydrocarbons from plant secondary metabolites. The former contains PCBs and likely low levels of dioxins or other angular ether structures due to industrial activity, and the latter could have been exposed to polyaromatic ethers from natural prairie fires. The PCR primers used in this study were synthesized with sequencing adapters and 8 base oligo multiplex sequencing barcodes. PCR products were prepared as described previously (Iwai et al., 2010) and pooled with other samples for pyrosequencing (Roche 454 GSFLX Titanium Sequencer).

Raw reads were filtered through barcode matching and quality filtered using the Ribosomal Database Project II (RDP-II) Pyro Initial Process tool (Cole et al., 2009) using a forward primer maximum mismatch of 2 and minimum length of 300 bp. Reads passing the initial filters were aligned and frameshift corrected and translated into protein sequences using the RDP FrameBot tool [http://fungene.cme.msu.edu/FunGenePipeline/, see also Wang et al., 2013)]. A FrameBot reference set was obtained using manual selection for a broad diversity of aromatic (including dioxin, dibenzofuran, carbazole, biphenyl, PAH, phenylpropionate, etc.) hydroxylating dioxygenase genes from the FunGene repository with an HMM bits saved score cutoff of 350. Protein reads with a length >100 amino acids and ≥30% identity to the nearest reference sequence were used for further analysis (Table 1). UCHIME v4.2.40 using the de novo mode was used to determine chimeric sequences. These quality filtered protein sequences and corresponding reference amino acid sequences were aligned by HMMER and trimmed. The obtained sequences were combined with reference sequences and were clustered at 50% dissimilarity, using the RDP mcClust tool (Cole et al., 2009). This clustering method was selected because there are so few known strains with activity toward dioxins. Clusters not containing a reference sequence were considered novel clusters (Iwai et al., 2010). The 50% dissimilarity cutoff was chosen as this approximate distance is where reference sequences were clustered to determine primer design groups. One representative sequence from each cluster was selected using the representative sequence tool on the RDP FunGene pipeline. These sequences were used to construct a nearest neighbor-joining tree using MEGA 5.1 software. No amplification products were obtained using the *dbfA1* primer set. Also eight *dxnA1*/*dfdA1* sequences were classified as chimeras by UCHIME; however, they were singleton sequences that were not considered in downstream analyses. Sequences were deposited in the European Nucleotide Archive under accession numbers ERS329748-ERS329751 and ERS329770-ERS329772.

**Table 1:** **Obtained sequences statistics**.

**Primer set**	**Sample name**	**Match barcode**	**Passed initial processing**	**Contains frame-shift**	**Passed Frame-Bot**	**Avg. length**
*dxnA1*/*dfdA1*	Rhizosphere	2,319	2,095	456	641	389
	KS	2,844	2,521	451	690	375
	Environmental sample total	5,163	4,616	907	1,331	
	MC	1,247	1,204	626	1,204	450
*carAa*	Rhizosphere	720	543	128	193	460
	KS	673	594	194	339	465
	Environmental sample total	1,393	1,137	322	532	
	MC	612	501	340	500	476

A mock community (MC) was composed of the strains used in validation of primer specificity and yielded the correct sequences and are not described further.

**Figure 1:**
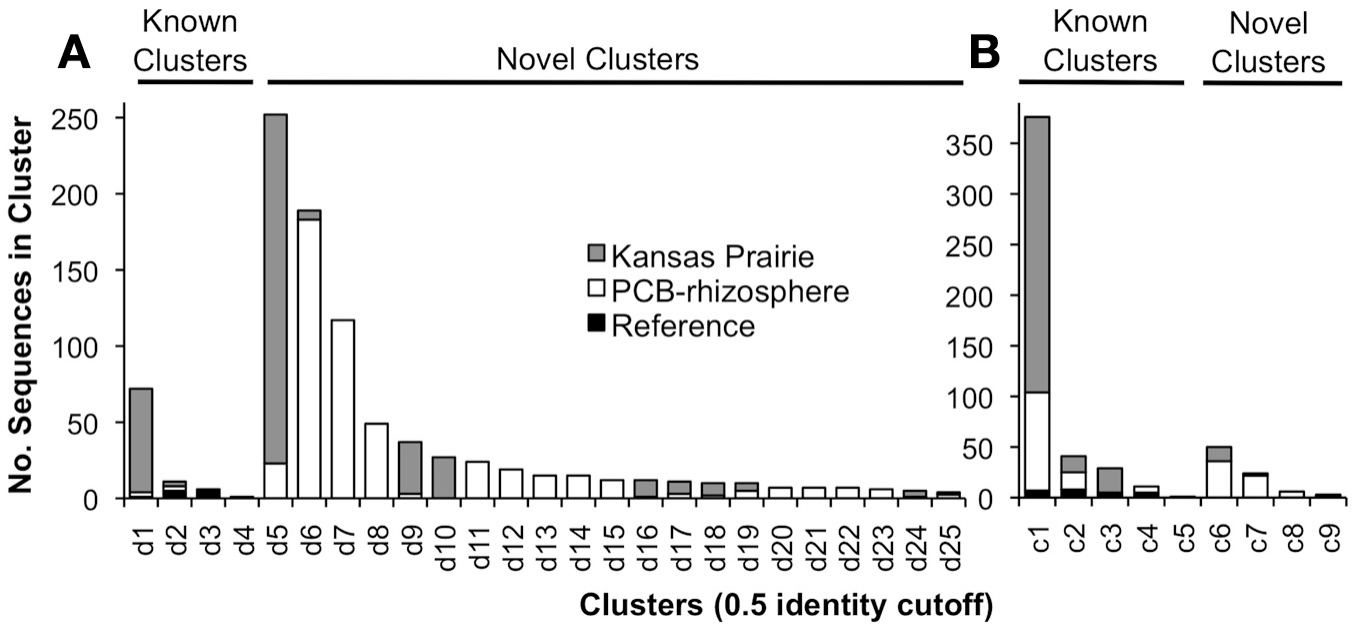
**Results of clustering obtained sequences with the reference sequences. (A)** Results using the *dxnA1*/*dfdA1* primer set. **(B)** Results using the *carAa* primer set. Clusters are only shown that contained at least four sequences. There were an additional 12 clusters that contained two or three sequences.

**Figure 2:**
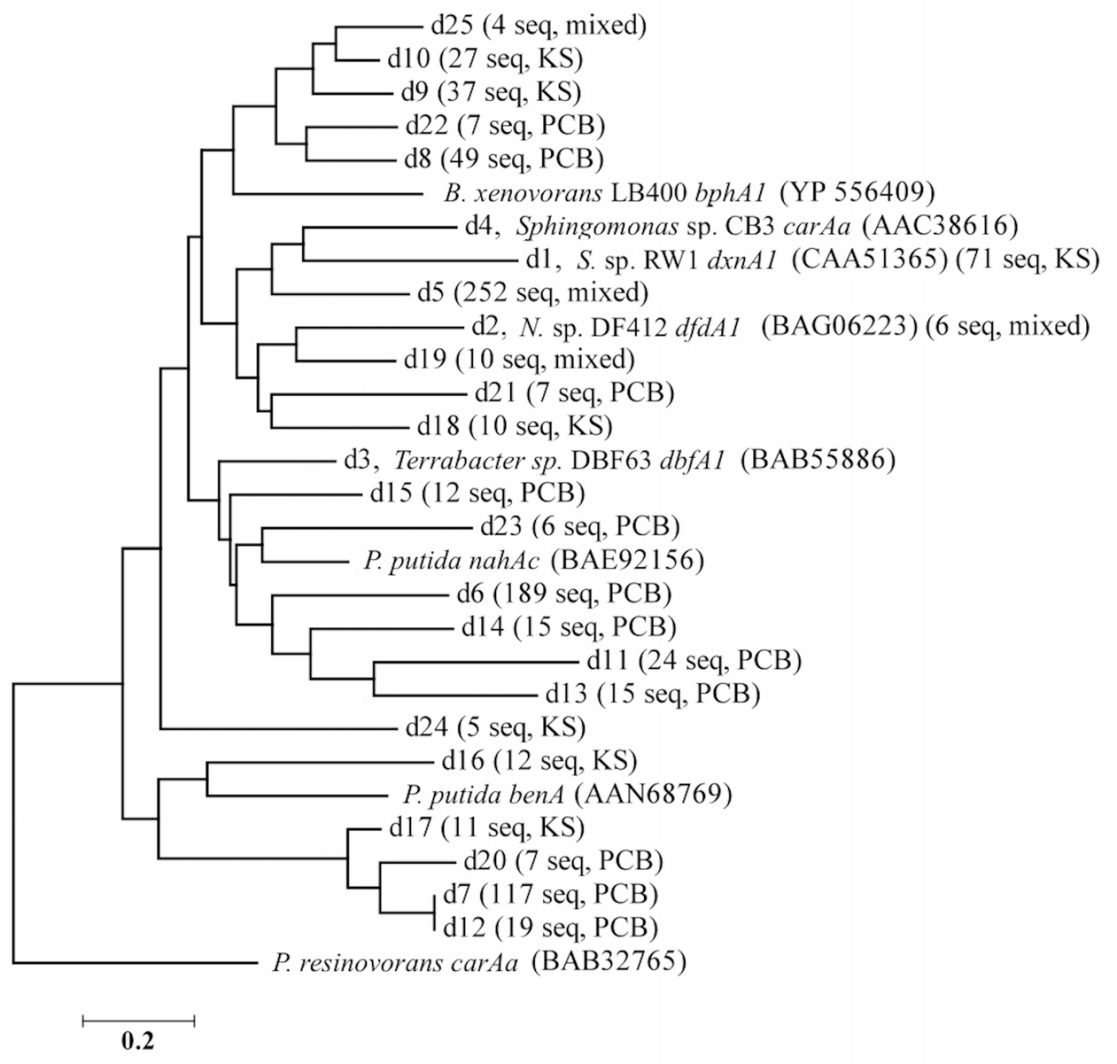
**Nearest neighbor-joining tree of the representative sequences of each *dxnA1* cluster shown in Figure 1**. Branch names designate: cluster name (from Figure 1), name and accession number of reference sequence in that cluster (if applicable), number of obtained sequences from pyrosequencing, and the predominate sample from which the sequences originated. Sequences were aligned using MUSCLE, trimmed to a common region for all sequences, and the tree was made using MEGA 5.1. N. sp. DF412 refers to Nocardioides sp. DF412.

The 1,863 obtained sequences revealed the *in situ* diversity of dioxygenases with suspected activity toward dioxins (Figure 1). The majority of *dxnA1*/*dfdA1* sequences formed novel clusters (Figure 1A). Many clusters were shared in both the KS soil and the PCB soil. However, the dominant *dxnA1*/*dfdA1* clusters differ between sites. Two clusters (clusters d1, d5) comprise 68% of sequences in the KS sample while these same clusters only represent 4% of the PCB soil sequences. Notably, cluster d1 contains the reference sequence from the (chloro)dioxin oxidizing *Sphingomonas wittichii* str. RW1 and represents nearly 10% of the KS soil obtained dioxygenase gene community. This indicates that genes similar to this important dioxygenase are present in this prairie soil. The PCB sample was dominated (49% of sequences) by two clusters, d6 and d7, which represent only 1% of the KS sequences. These data show site-specific populations having novel dioxygenases. The specificity of the primer sets was re-affirmed as reference sequences used to design the *dbfA1* primer set did not cluster with any of the obtained sequences using the *dxnA1*/*dfdA1* primer set at 50% dissimilarity. Similarly, many of the obtained sequences, including the most abundant novel cluster, formed a clade on the same branch as *dxnA1* and *dfdA1* (Figure 2). Other sequences were more distantly related to this clade, which may denote differing dioxygenase specificities. While a majority of *carAa* sequences clustered with reference sequences at 50% dissimilarity (Figure 1B, cluster c1), similar ecological trends, including site-specific populations, were also observed within the *carAa* sequences. The *carAa* obtained sequences cluster separately from the reference sequences at 70% similarity cutoff.

### Sequence conservation

**Figure 3:**
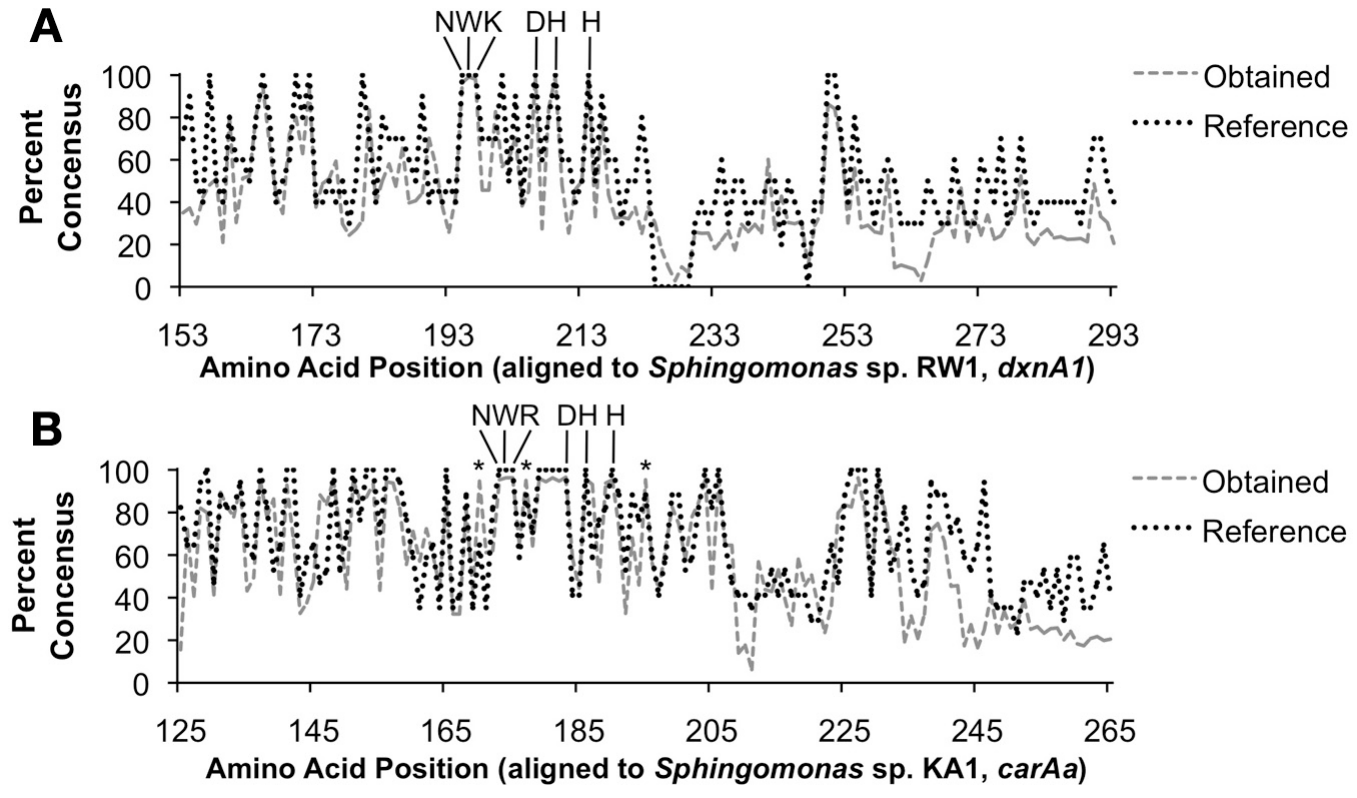
**Percent conservation of translated obtained nucleotide sequences to protein sequences. (A)** Results using the *dxnA1*/*dfdA1* primer set. **(B)** Results using the *carAa* primer set. Key conserved amino acid positions are indicated. The DX_2_HX_3−4_H iron-binding site is indicated as well as the uncharacterized, yet highly conserved NW(K/R) motif. The asterisk (^*^) indicates positions for which obtained sequences were conserved at a higher rate than reference sequences.

A consensus amino acid sequence based on the obtained sequences was compared to an aligned consensus sequence of reference (GenBank) sequences. When we searched for conserved amino acids, a known iron-binding motif (DX_2_HX_3−4_H (Nojiri et al., 2005), where X is any given amino acid) was observed in >95% of sequences of both *dxnA1*/*dfdA1* and *carAa* obtained sequences (Figure 3). In addition, another conserved motif (>95%), NWK or NWR, was observed. Although no associated function could be found in the literature regarding a role of the highly conserved NW(K/R) motif including a search of the characterized *carAa* protein structure (Nojiri et al., 2005), the motif appears essential to the protein. It is possible that the NW(R/K) motif plays a role in positioning of the substrate binding amino acid at the active site of the protein. In the case of *carAa*, Gly-178 is implicated to hydrogen bond to carbazole and the NWR motif is situated on the same alpha helix as Gly-178 (Nojiri et al., 2005). The identity of the third amino acid of this motif is specific to each group.

While some recent advancements have been made in dioxin degradation with previously isolated strains, especially *S*. *wittichii* str. RW1 (Nam et al., 2006), progress in isolating novel degrading strains has been slow for several decades (Field and Sierra-Alvarez, 2008). In the neighboring biphenyl dioxygenase clade, despite having many more degrader strains isolated, gene-targeted metagenomics still revealed extensive novel diversity (Iwai et al., 2010). For dioxin clades, amplicon pyrosequencing revealed novel dioxygenase sequence clusters of intermediate sequence similarity between the *dxnA1* and *dfdA1* genotypes. This reveals a likely continuum of genetic diversity between these two distinct but functionally similar groups. According to obtained dioxygenase sequences, the majority of potential dioxin degraders in these communities have no cultured representative, and their diversity, in terms of cluster abundance, far exceeds that of known degraders, as was previously found for *bphA1* (Iwai et al., 2010).

## *nirS* and *nirK* primer coverage and clustering

Denitrifiers are an important functional group in the nitrogen (N) cycle and N-cycle functional genes have been targeted for diversity, abundance and expression studies (Braker et al., 2000; Prieme et al., 2002; Philippot et al., 2007; Palmer and Horn, 2012). However, primer coverage poses a continuing problem in the amplification and sequencing of N cycle functional genes in environmental samples. The majority of the current primers are based on the alignment of relatively few reference genes and tested on a relatively small number of type strains. For example, the majority of *nirK* primers are based on class I CuNIR genes from α-proteobacteria (Braker et al., 1998; Jones et al., 2008) and do not amplify class II and III *nirK* sequences (Green et al., 2010), which include the archaeal *nirK* (Treusch et al., 2005; Bartossek et al., 2010). Overall, few have investigated coverage of these primers *in-silico* (Throbäck et al., 2004; Heylen et al., 2006; Green et al., 2010) with comparison to environmental datasets. Current *nirS* and *nirK* primer limitations are thus due, in part, to the limited number of functional gene sequences from cultivated and identified denitrifiers (Heylen et al., 2006), high sequence divergence at current primer sites (Green et al., 2010), and the availability of but a few deep sequencing studies (Palmer et al., 2012; Palmer and Horn, 2012) that can be used to gauge primer performance in environmental matrices.

### Initial sequence processing

**Figure 4:**
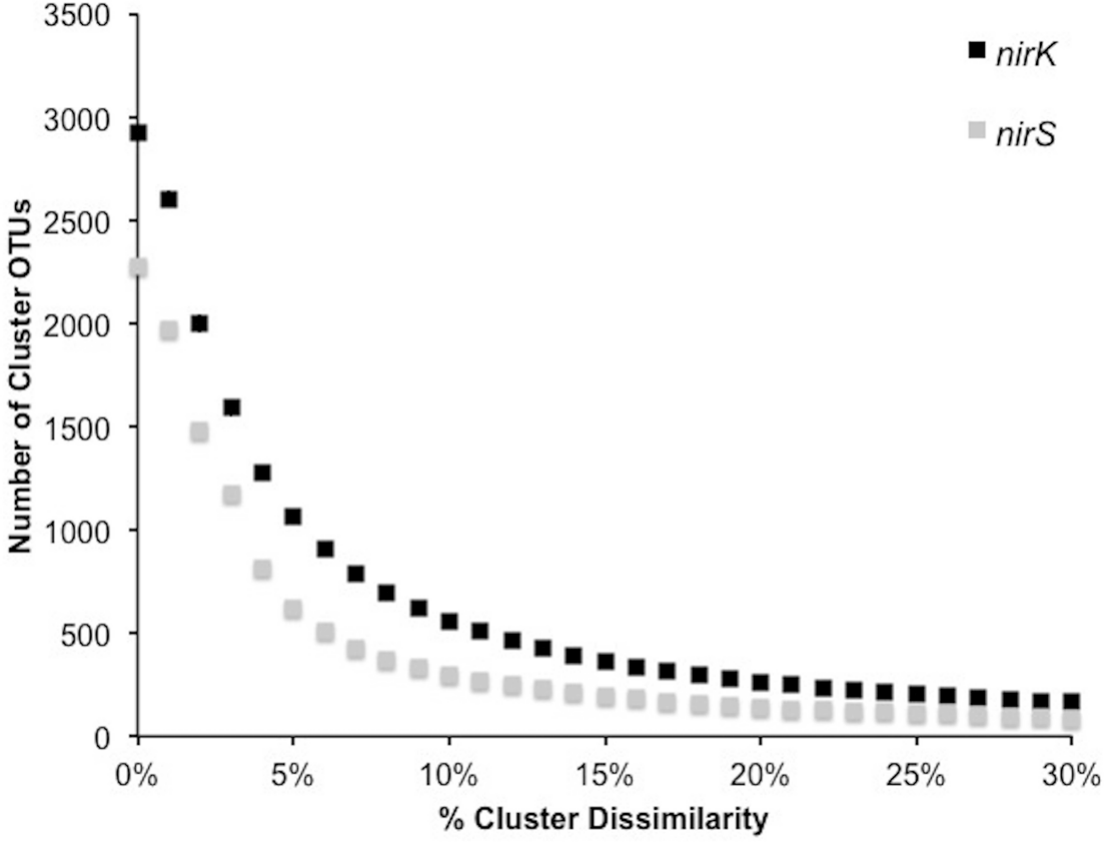
**Number of OTUs generated by complete linkage clustering of aligned protein sequences for *nirK* and *nirS* from 0 to 30% dissimilarity**.

In order to investigate the diversity of denitrifiers and test primer coverage in environmental samples *nirK* was amplified using primers nirK517F/1055R (Chen et al., 2010) and *nirS* with cd3af (Michotey et al., 2000) and R3cd (Throbäck et al., 2004) with 9 bp tag sequences using DNA extracted from six tallgrass prairie sites (34°58′54″N, 97°31′14″W) using freeze-grinding mechanical lysis (Zhou et al., 1996). Sequences were deposited in the European Nucleotide Archive under accession numbers ERS329737-ERS329747. Raw sequences were processed through the RDP pyrosequencing pipeline and the ~540 and ~425 bp amplicons yielded sequences averaging 456±45 bp (*nirK*) and 383±36 bp (*nirS*). Since the forward and reverse reads overlapped each other, both directions were analyzed together (Palmer and Horn, 2012; Palmer et al., 2012). Translation and frameshift correction were performed using the RDP FrameBot tool. The average number of frameshifts per gene was nearly identical (*nirK* 1.0 ± 1.3, *nirS* 0.6 ± 1.0) with the model alignment covering the full amplicon length. Complete linkage clustering on 3,400 randomly resampled aligned protein sequences from six replicates per gene revealed an inflection point of approximately 5–8%, reflecting a change from intra- to inter-species sequence homology (Figure 4). A 5% protein-protein dissimilarity was chosen for both genes for downstream analyses. This is in contrast to previous studies where *nirK* and *nirS* were clustered at 4% nucleotide dissimilarity (Chen et al., 2010) or *nirK* at 17% and *nirS* at 18% (Palmer et al., 2012; Palmer and Horn, 2012). A mean amino acid dissimilarity of 5% of genes in common in genomes corresponds to the 70% DNA-DNA hybridization that is currently the definition of bacterial species. Hence a 5% clustering cutoff approximates species level differences (Cole et al., 2010).

### *nirK* reference dataset and primer coverage

For *nirK*, 523 sequences from the RDP FunGene database were chosen as a BLASTp reference database using 50% minimum HMM coverage and 100 minimum score filters followed by further database refinement. This included 69 sequences linked to unclassified taxa and 114 linked to unique genera comprising 215 unique species. Blastp of the 1,068 representative cluster sequences at 5% protein-protein dissimilarity yielded only 52 unique closest-match species comprising 20 classified genera (Table 2). Previously, with a pyrosequencing depth of 11,612 sequences, *nirK* was assigned to 9–10 species-level OTUs in palsa peat and permafrost soil samples using a 17% dissimilarity threshold (Palmer and Horn, 2012; Palmer et al., 2012).

**Table 2:** **Closest BLASTp hits for *nirK* cluster representative sequences at the genus level with the average BLASTp identity with standard errors and the range of percent identities for each genus**.

**Genus**	**Avg % ID**	**% ID Range**
*Achromobacter*	81.5 ± 0.3	42.1 – 100
*Afipia*	83.4 ± 0.3	71.9 – 94.0
*Alcaligenes*	77.3 ± 0.4	67.1 – 86.1
*Bradyrhizobium*	82.6 ± 0.2	56.5 – 99.4
*Chelativorans*	81.1 ± 1.2	66.1 – 88.9
*Maritimibacter*	73.4 ± 2.2	68.9 – 75.8
*Mesorhizobium*	79.8 ± 0.2	62.1 – 100
*Methylocystis*	84.7 ± 1.2	80.6 – 90.4
*Nitratireductor*	82.8 ± 0.3	65.8 – 97.3
*Nitrosomonas*	70.3	n.d.
*Ochrobactrum*	92.8 ± 0.4	69.1 – 97.7
*Phaeobacter*	71.9	n.d.
*Pseudomonas*	87.6 ± 2.6	79.0 – 97.7
*Rhizobium*	85.6 ± 2.1	75.3 – 95.7
*Rhodopseudomonas*	80.6 ± 0.1	49.5 – 92.2
*Roseovarius*	67.1	n.d.
*Shewanella*	73.2 ± 1.4	68.2 – 76.3
*Sinorhizobium*	88.4 ± 1.4	70.4 – 99.4
*Starkeya*	87.2 ± 3.2	68.4 – 93.6
Uncultured bacteria	82.5 ± 1.0	52.9 – 98.8

In order to understand this low retrieved *nirK* diversity, we analyzed the role of primer coverage by using the updated *nirK* reference database with RDP probe match. The *nirK517F* primer was specific for 96 strains comprising 39 species, 145 with 1 mismatch, and 188 with 2 mismatches while *nirK1055R* was specific for 46 strains composed of 17 species, 104 with 1 mismatch and 121 with 2 mismatches. Other frequently used *nirK* primers such as F1aCu/R3Cu (Throbäck et al., 2004) and nirK1F/nirK5R (Braker et al., 1998) were also evaluated (Table A3). All primers preferentially targeted the α-Proteobacteria, although captured diversity will likely be limited by reverse primer sequence homology when invoking strict PCR conditions. This issue was not evident in our results. Only the *Achromobacter* and *Alcaligenes* were captured with 0 mismatches, leaving 14 genera in the β-Proteobacteria. Likewise, only the *Pseudomonas* and *Shewanella denitrificans* of the 23 γ-Proteobacteria reference genera were hit. The remaining 83 species from the class II and III *Archaea*, *Actinobacteria*, *Chloroflexi-Firmicutes* and *Bacteroidetes* were not targeted by any primer. Overall, primer coverage of the reference dataset was extremely low, resulting in a constrained diversity that re-affirms an urgent need for further exploration of *nirK* primer design, perhaps to other primer-binding regions (Green et al., 2010).

### *nirS* clustering and primer coverage

**Figure 5:**
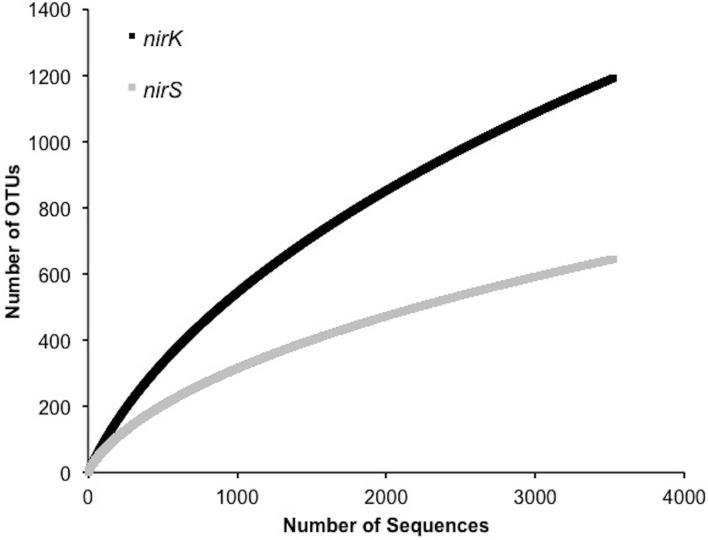
**Rarefaction curves for 5% OTU dissimilarity for *nirK* and *nirS***.

A significantly lower number of classified, non-environmental sequences are available for *nirS*. Using a minimum HMM coverage of 50 and score of 600, a total of 109 *nirS* reference sequences comprising 45 unique genera and 63 species were obtained from the FunGene database for both taxonomic assignment and primer analyses. BLASTp of the 617 representative sequences at 5% protein-protein dissimilarity yielded 18 unique clusters linked to classified genera comprising 24 unique species and 3 unclassified bacteria (Table 3). Previously, 14 OTUs of *nirS* were retrieved from 918 sequences using an 18% nucleotide dissimilarity (Palmer and Horn, 2012). Overall, *nirS* total diversity was more constrained than *nirK*, as indicated by rarefaction curves (Figure 5). Average identity to the reference database was lower for *nirS* (76.5 ± 0.2%) than for *nirK* (81.7 ± 0.1%), similar to the lower nucleotide identity of *nirS* (74.7%) to the GenBank database than *nirK* (90.7%) in a previous study (Chen et al., 2010).

**Table 3:** **Closest BLASTp hits for *nirS* cluster representative sequences at the genus level with the average BLASTp identity with standard errors and the range of percent identities for each genus**.

**Genus**	**Avg % ID**	**% ID Range**
*Aromatoleum*	74.3 ± 0.5	73.9 – 74.8
*Azoarcus*	72.4 ± 0.9	70.8 – 73.7
*Brachymonas*	73.4 ± 3.8	65.9 – 77.9
*Cupriavidus*	78.1 ± 0.3	67.2 – 99.2
*Dechloromonas*	96.6 ± 0.0	96.6 – 96.6
*Dinoroseobacter*	75.3 ± 2.8	72.3 – 80.9
*Kangiella*	72.0 ± 0.2	70.3 – 73.9
*Magnetospirillum*	77.0 ± 0.9	70.2 – 90.2
*Marinobacter*	71.0 ± n.d.	n.d.
*Polymorphum*	69.5 ± n.d.	n.d.
*Pseudogulbenkiania*	68.7 ± n.d	n.d.
*Pseudomonas*	76.8 ± 0.7	68.9 – 93.1
*Ralstonia*	76.5 ± 0.1	72.7 – 80.0
*Rubrivivax*	83.2 ± n.d.	n.d.
*Ruegeria*	73.3 ± 0.7	69.1 – 77.5
*Sideroxydans*	80.5 ± 1.1	79.4 – 81.7
*Stappia*	74.2 ± 0.7	69.9 – 76.9
*Thiobacillus*	72.2 ± 0.5	68.9 – 75.4
Uncultured bacteria	76.6 ± 0.3	68.0 – 94.4

For the primer analyses, primer cd3af hit 62 strains comprising 28 unique species with 0 mismatches, 73 with 1 and 89 with 2 mismatches within the reference dataset. Primer R3cd hit 48 strains comprising 27 unique species with 0, 67 strains with 1 and 98 with 2 mismatches. Another primer set (nirS1F/nirS6R; Braker et al., 1998) was also evaluated (Table A4). Primers nirS1F/nirS6R exhibited better coverage of the α-Proteobacteria while both were comparable in the β- and γ-Proteobacteria taxa. Neither performed well in targeting the *Chloroflexi*, *Deinococcus-Thermus*, *Aquificales* or *Bacteroidetes*. In both instances coverage was highly dependent on PCR stringency, with near full coverage of the β-Proteobacteria at 2 primer mismatches. Unlike *nirK*, refinement through degeneracy of the current primer sets should allow for higher coverage, although the current sequence availability from classified strains remains low.

## *nifH* sequences processed using supervised and non-supervised methods

When pyrosequencing data are used to compare gene profiles or gene diversities among samples, it is necessary to first bin the sequences by one of two general methods. Either sequences can be clustered into OTUs at a specified distance (the unsupervised method) or sequences may be classified directly using a reference database [the supervised method, as in Wang et al. (2013)]. The choice of method depends on the specific goals and, to some extent, the current knowledge of the target gene. Clustering better preserves information on diversity and better enables the discovery of novel gene sequences while the supervised method yields more immediately interpretable results and better enables comparisons between different experiments. It is expected to fail, however, in instances where the reference database captures little of the existing gene diversity.

To contrast the performance of the supervised and unsupervised methods, soil samples were chosen from an investigation of various cropping systems on microbial soil diversity. These samples came from soils under corn, switchgrass and prairie species and represent the range of soil types in central to southern Michigan and Wisconsin. *NifH* sequence libraries were produced from DNA extracted from these soil samples by PCR per the protocol described by Wang et al. (2013) and analyzed for differences in gene diversities and gene profiles after binning sequences by each method.

Primer design is critical to capturing diversity of any gene (Iwai et al., 2011a). For nitrogen fixation, primers for *nifH* have been recently evaluated *in silico* (Gaby and Buckley, 2012) and the Zf/Zr (Zehr and McReynolds, 1989) primer combination was found to have high theoretical performance, matching 92% of all reference sequences including all *nifH* groups I, II, and III, versus 25% for the PolF/PolR (Poly et al., 2001) primers. However, the Zf/Zr combination proved impractical in use, giving non-specific products and smeared bands on gels when used to amplify DNA extracted from soil (Gaby and Buckley, 2012). Better performing primer combinations, such as those identified by Gaby and Buckley, should be evaluated for future pyrosequencing studies taking into consideration coverage of groups important to the habitat studied.

Because they more reliably amplify DNA extracted from soil, primers PolF and PolR (Poly et al., 2001) were used in this study. These primers target an approximately 320 bp region of the *nifH* gene. The forward primer consisted of the 25 bp 454 A Adapter, a 10 bp barcode, followed by the 20 bp primer PolF (5′-CGT ATC GCC TCC CTC GCG CCA TCA G-barcode-TGC GAY CCS AAR GCB GAC TC-3′). The reverse primer consisted of the 25 bp 454 B Adapter and the 20 bp primer PolR (5′-CTA TGC GCC TTG CCA GCC CGC TCA GAT SGC CAT CAT YTC RCC GGA-3′). PolF and PolR are similar to Zf and Zr (Zehr and McReynolds, 1989) which we also considered using, but were modified to be less degenerate while maintaining broad coverage of *nifH* cluster I. When originally tested, they captured all 19 test strains, but these were limited to α-, β-, and γ-Proteobacteria, Actinobacteria, and Firmicutes (Poly et al., 2001). When tested with DNA extracted from pasture and cornfield soils, these primers produced bands of the expected size that hybridized *nifH* probe from *Azospirillum*, and did not produce non-specific products.

Initial processing of the pyrosequencing reads was performed using tools available on the Ribosomal Database Project's (RDP) FunGene pipeline web site. After reads were quality filtered and barcode sorted, FrameBot was used for translation and frame shift correction by comparing sequences to those in a reference data set containing 782 unique sequences trimmed to cover the nifH amplicon region. Sequences were deposited in the European Nucleotide Archive under accession numbers ERS329752-ERS329769.

Sequencing data was processed by closest match analyses and by clustering at a 5% distance, and analyzed using the packages vegan (Oksanen et al., 2013) and phyloseq (McMurdie and Holmes, 2012) in R (R Core Team, 2013). In both cases, the number of sequences was rarefied to the minimum number of sequences per sample and empty OTUs removed. In the case of closest match, this left 3,693 sequences per sample in 160 OTUs representing 83 genera. In the case of clustering, this left 3,750 sequences per sample in 1,706 OTUs representing 81 genera.

By far, the majority of sequences were identified as Proteobacteria, further classified to α-, β-, γ-, and δ-Proteobacteria. The primers were originally designed to amplify *nifH* sequences from Proteobacteria, Firmicutes, and Actinobacteria, but a significant number of Verrucomicobia sequences were obtained as well (Table 4). Approximately 4% of the sequences were similar to environmental sequences that could not be classified to the phylum level, and may therefore represent novel sequences.

**Table 4:** **Distribution of *nifH* sequences recovered from Michigan and Wisconsin prairies and sites cultivated with corn and switchgrass**.

**Phylum**	**No. sequences**	**%**
Proteobacteria	80,233	91.619
α-Proteobacteria	35,850	40.954
β-Proteobacteria	19,558	22.236
γ-Proteobacteria	1,871	2.107
δ-Proteobacteria	22,954	26.203
Cyanobacteria	2,003	2.287
Verrucomicrobia	1,134	1.295
Firmicutes	281	0.321
Actinobacteria	124	0.142
Nitrospirae	94	0.107
Spirochaetes	72	0.082
Bacteroidetes	17	0.019
Chlorobi	10	0.011
Euryarchaeota	6	0.007
Fusobacteria	1	0.001
Environmental samples^*^	3,597	4.107

* The reference, AF194084.1, an environmental sequence, shares 97% identity with the gene from Azospirillum sp. B510 (YP_003447953.1).

**Figure 6:**
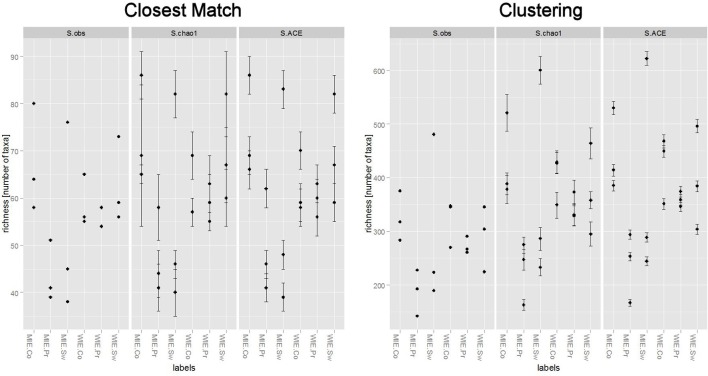
**Differences in the number of *nifH* species observed and estimated between closest match (supervised method) and clustering (unsupervised method)**.

**Figure 7:**
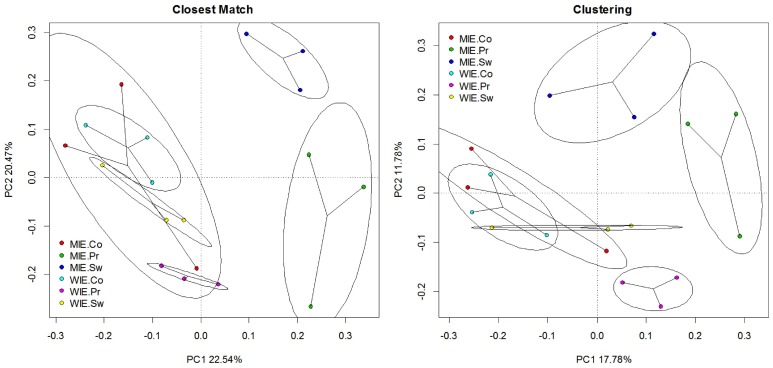
**Principal component analysis of Hellinger transformed *nifH* data (square root of relative abundance) on closest match and clustering data illustrating changes in relationships among sites**.

Unsurprisingly, a greater number of OTUs are observed and estimated when sequences are clustered (Figure 6). Comparisons among treatments, however, are similar. Clustering better resolves samples by estimated number of species; that is, standard errors are relatively smaller. Ordinations of data resulting from closest match and clustering are generally similar with the Michigan prairie and Michigan switchgrass sites separated from each other and from the other sites using both methods (Figure 7). In this case, the clustering based analysis provides greater resolution as it also separates Wisconsin prairie sites from the others.

Multiple *F*-tests were performed for difference in taxa abundance among treatments for data processed by both means. For the closest match method, 12 OTUs were found with an unadjusted *p* < 0.05, but none were significant after correcting for false discovery rate. For the clustering method, 46 OTUs were found with an unadjusted *p* < 0.05, and one of these was significant with adjusted *p* < 0.05. Clustr0103, genus *Methylosinus*, occurred exclusively in corn samples and was more abundant at the Michigan ones.

In the case of *nifH* presented here, the supervised and unsupervised methods provide similar results. This is because the database was tailored to *nifH* sequences amplifiable by the primer combination PolF/PolR and does capture most of the gene diversity in the amplicon libraries. For that reason, relatively few sequences are distant from their closest match in the database used for their identification. When this is not the case, identification to closest match may be binned into subcategories by separate bins encompassing those >90% similar to closest match, 75–90%, 50–75% similar, and those less than 50% similar. This binning by distance minimizes binning disparate sequences and is to be preferred for that reason. As an aid to interpretation, taxonomy may be assigned to clusters using a similar scheme.

Even though the difference in performance between the two methods, supervised and unsupervised, was minimal for this data set, clustering provided better estimates of total diversity, and proved more powerful in resolving differences in structure between treatments and in finding significantly different OTUs among treatments. For these reasons, it is recommended as the preferred method, and especially so when the reference database is less comprehensive than the one for *nifH*, which is currently the case for virtually all ecofunctional genes.

## Conclusions

Amplicon functional gene sequencing provides an important companion method, and possibly in the future an alternative, provided full-length open reading frames can be obtained, to traditional strain isolation to discover desired novel genes for biotechnology. In their effect on diversity estimates and community changes with treatment, these studies illustrate that current primer coverage and specificity remain key issues that should be addressed with a robust, curated reference database. Highly divergent gene functional groups will probably need to be targeted with multiple primer sets and/or at other conserved regions, as is the case with *nirK*. Compared to the direct classification of sequences, clustering is the preferred method, resulting in higher estimates of total diversity and better resolution between treatments. However, estimates of community structure and differences between treatments are also impacted by the use of highly variable clustering dissimilarities. As such, the properties of each gene and the coverage of the accompanying reference database should be considered prior to formulating the downstream sequence processing methodology. Protein-coding genes, by their nature, are more varied in their sequences than rRNA genes where it is the primary structure that is constrained. Thus to expect a single degenerate primer set for a functional gene to be comprehensive is unrealistic. However, by using well-defined conditions and constant, well-performing primers, a standard subset of nature's functional guilds can be recovered and comparative studies can be done. Rarely in microbial ecology can one be comprehensive, and ecofunctional gene analysis is no exception. As with other microbial ecology studies, however, useful knowledge can still be gained with the understanding of constraints in interpretations.

### Conflict of interest statement

The authors declare that the research was conducted in the absence of any commercial or financial relationships that could be construed as a potential conflict of interest.

## Appendix

**Table A1:** **Reference sequences used in primer design, PCR validation of primer specificity and designation of reference sequences in clusters with obtained environmental sequences**.

**Cluster no**.	**Organism (Protein ID)**	**Primer group**	**PCR validation**	**References**
d1	*S. wittichii* str. RW1 (CAA51365)	1	*dxnA1*	Wittich et al., 1992
d2	*Terrabacter sp.* YK3 (BAC06602)	1		Iida et al., 2002a
d2	*Nocardioides* sp. DF412 (BAG06223)	1	*dxnA1*	Miyauchi et al., 2008
d2	*Rhodococcus* sp. HA01 (ACC85677)	1		Aly et al., 2008
d2	*Terrabacter* sp. YK1 (BAG80728)	1		Iida et al., 2002a
d2	*Rhodococcus* sp. YK2 (BAG80733)	1	*dxnA1*	Iida et al., 2002b
d3	*Bacillus tusciae* DSM 2912 (YP_003590130)			
d3	*Terrabacter* sp. DBF63 (BAB55886)	2	*dbfA1*	Kasuga et al., 2001
d3	*Rhodococcus* sp. HA01 (ACC85681)	2		Aly et al., 2008
d3	*Sphingomonas* sp. LB126 (ABV68886)	2		Schuler et al., 2008
d3	*Paenibacillus* sp. YK5 (BAE53401)	2	*dbfA1*	Iida et al., 2006
d3	*Rhodococcus* sp. YK2 (BAC00802)	2	*dbfA1*	Iida et al., 2002b
d3	*Rhodococcus* sp. DFA3 (BAD51811)		*dbfA1*	Noumura et al., 2004
d4	*Sphingomonas* sp. CB3 (AAC38616)	1		Shepherd and Lloyd-Jones, 1998
NA	*Sphingomonas* sp. KA1 (YP_718182)	2		Urata et al., 2006
c1	*Sphingomonas* sp. KA1 (YP_717942)	3	*carAa*	Urata et al., 2006
c1	*Sphingomonas* sp. JS1 (ACH98389)	3		
c1	*Sphingomonas* sp. KA1 (YP_717981)		*carAa*	
c1	*Sphingomonas* sp. XLDN2-5 (ADC31794)			
c4	*Pseudomonas stutzeri* OM1 (BAA31266)	3	*carAa*	Ouchiyama et al., 1998
c4	*P. resinovorans sp.* CA10 (NP_758566)	3	*carAa*	Ouchiyama et al., 1993
c4	*Janthinobacterium* sp. J3 (BAC56742)	3	*carAa*	Nojiri et al., 2005
c4	*Pseudomonas* sp. XLDN4-9 (AAY56339)	3		Li et al., 2004
c4	carbazole-degrading bacterium CAR-SF (BAG30826)	3		Fuse et al., 2003
c4	*Pseudomonas* sp. K23 (BAC56726)			
c5	*Nocardioides* sp. IC177 (BAD95466)	3		Inoue et al., 2005
NA	*Burkholderia xenovorans* LB400 *bphA1*	none	none	
NA	*Rhodococcus* sp. RHA1 *bphA1*	none	*dbfA1*^*^	

PCR validation column indicates which strains were used as PCR positive controls, and which primer set produced an amplicon with that strain. References listed detail the activity of the strain toward dioxins.

* Rhodococcus sp. RHA1 produced only a faint band with the dbfA1 primer set.

**Table A2:** **Primer sequences and PCR conditions of the three primer sets**.

**Primer Set**	**Target position**	**Sequence (5′-3′)**	**Ta^‡^ (°C)**	**Primer conc. (μM)**	**Mg^2+^ conc. (mM)**
*dxnA1*/*dfdA1*	145-150^*^	TACAAVGGGCTGRTTTTCGG	51	1.2	4
	312-307^*^	GARAAVTTVGGGAACAC			
*dbfA1*	205-210^*^	GGCGACGACTAYCACGTGCT	51	0.8	3.5
	373-368^*^	TCGAAGTTCTCGCCRTCRTC			
*carAa*	69-74^†^	TGCCTNCAYCGHGGBGT	63	0.8	2.5
	268-263^†^	TTSAGHACRCCBGGSAGCCA			

These PCR conditions were optimized for the soil samples described. The target positions described are for reference amino acid sequences:

* position based on Sphingomonas wittichii RW1, dxnA1, and

† position based on Sphingomonas sp. KA1, carAa.

‡ annealing temperature.

**Table A3:** **Primer hits for 517F/1055R, F1aCu/R3Cu, nirK1F/nirK5R with 0 mismatches to the *nirK* dataset**.

	**517F**	**1055R**	**F1aCu**	**R3Cu**	**nirK1F**	**nirK5R**
**ALPHA-PROTEOBACTERIA**									
Afipia sp.	x					
Agrobacterium tumefaciens			x		x	
Azospirillum brasilense						
Azospirillum lipoferum						
Azospirillum sp.						
Bradyrhizobium japonicum	x					
Bradyrhizobium sp.	x					x
Brucella abortus	x				x	
Brucella canis	x				x	
Brucella ceti	x				x	
Brucella melitensis	x				x	
Brucella microti	x				x	
Brucella ovis	x				x	
Brucella pinnipedialis	x				x	
Brucella sp.	x	x			x	
Brucella suis	x				x	
Caulobacter segnis						
Chelativorans sp.	x	x				
Hyphomicrobium denitrificans						
Maritimibacter alkaliphilus	x	x				x
Mesorhizobium alhagi	x					
Mesorhizobium amorphae	x					
Mesorhizobium australicum						
Mesorhizobium ciceri	x					
Mesorhizobium opportunistum	x					
Methylocella silvestris						
Methylocystis sp.	x					
Nitratireductor aquibiodomus	x		x			
Nitrobacter hamburgensis						
Nitrobacter sp.						
Nitrobacter winogradskyi						
Ochrobactrum anthropi	x	x	x	x	x	x
Ochrobactrum intermedium	x	x	x	x	x	x
Oligotropha carboxidovorans						
Parvibaculum lavamentivorans						
Phaeobacter gallaeciensis			x			
Phenylobacterium zucineum						
Rhizobium etli	x		x		x	
Rhizobium sullae	x	x	x			x
Rhodobacter sphaeroides	x	x	x	x		x
Rhodopseudomonas palustris	x	x	x			
Rhodopseudomonas sp.	x	x				
Roseobacter sp.			x			
Roseovarius sp.						
Sinorhizobium fredii	x	x	x		x	
Sinorhizobium medicae	x	x			x	
Sinorhizobium meliloti	x		x		x	x
Sinorhizobium sp.			x		x	
Sphingomonas wittichii						
Starkeya novella	x	x				x
**BETA-PROTEOBACTERIA**									
Achromobacter cycloclastes			x		x	
Achromobacter sp.					x	x
Achromobacter xylosoxidans	x	x		x	x	
Alcaligenes faecalis	x	x	x	x	x	x
Alcaligenes sp.	x	x			x	
Azoarcus sp.						
Burkholderia mallei						
Burkholderia pseudomallei						
Burkholderia thailandensis						
Chromobacterium violaceum						
Herminiimonas arsenicoxydans						
Kingella denitrificans						
Kingella kingae						
Kingella oralis						x
Lautropia mirabilis						
Methylotenera mobilis						
Neisseria bacilliformis						
Neisseria cinerea						
Neisseria elongata						
Neisseria flavescens						
Neisseria gonorrhoeae						
Neisseria lactamica						
Neisseria macacae						
Neisseria meningitides						
Neisseria mucosa						
Neisseria polysaccharea						
Neisseria sicca						
Neisseria sp.						
Neisseria subflava						
Neisseria weaveri						
Nitrosomonas europaea						
Nitrosomonas eutropha						
Nitrosomonas sp.						
Nitrosospira briensis						
Nitrosospira multiformis						
Nitrosospira sp.						
Polaromonas naphthalenivorans						
Pusillimonas sp.	x					
Ralstonia pickettii						
Ralstonia solanacearum						
Ralstonia sp.						
Taylorella asinigenitalis						
Taylorella equigenitalis						
Bdellovibrio bacteriovorus						
**GAMMA-PROTEOBACTERIA**									
Cardiobacterium hominis						
Cardiobacterium valvarum						
Gallibacterium anatis						
Haemophilus parahaemolyticus						
Haemophilus parainfluenzae						
Haemophilus paraphrohaemolyticus						
Haemophilus pittmaniae						
Idiomarina loihiensis						
Kangiella koreensis						
Mannheimia succiniciproducens						
Marinobacter sp.						
Methylomonas sp.						
Moraxella catarrhalis						
Nitrococcus mobilis						
Nitrosococcus halophilus						
Nitrosococcus oceani						
Oceanimonas sp.						
Pasteurella bettyae						
Pseudoalteromonas haloplanktis						
Pseudomonas aeruginosa						x
Pseudomonas chlororaphis	x				x	
Pseudomonas entomophila			x			x
Pseudomonas fluorescens	x	x			x	
Pseudomonas mendocina	x	x				x
Pseudomonas sp.			x	x	x	
Pseudomonas stutzeri						
Pseudoxanthomonas suwonensis						
Psychrobacter sp.						
Rhodanobacter fulvus						
Rhodanobacter sp.						
Rhodanobacter spathiphylli						
Rhodanobacter thiooxydans						
Salinisphaera shabanensis						
Shewanella amazonensis						
*Shewanella denitrificans*	x					x
Shewanella loihica						
Shewanella woodyi						
Thioalkalivibrio sp.						
**BACTEROIDETES**									
Flavobacteriaceae bacterium						
Flavobacterium columnare						
Flavobacterium johnsoniae						
Maribacter sp.						
Marivirga tractuosa						
Rhodothermus marinus						
Belliella baltica						
Muricauda ruestringensis						
Aequorivita sublithincola						
Solitalea Canadensis						
Bizionia argentinensis						
Capnocytophaga gingivalis						
Capnocytophaga sp.						
Capnocytophaga sputigena						
Chryseobacterium gleum						
Imtechella halotolerans						
Myroides odoratimimus						
**ARCHAEA**									
Candidatus caldiarchaeum						
Candidatus nitrosoarchaeum						
Candidatus nitrosopumilus						
Haloarcula hispanica						
Haloarcula marismortui						
Haloferax denitrificans						
Haloferax lucentense						
Haloferax mediterranei						
Haloferax volcanii						
Halogeometricum borinquense						
Halomicrobium mukohataei						
Halopiger xanaduensis						
Halorhabdus utahensis						
Haloterrigena turkmenica						
Natrinema pellirubrum						
Natronomonas pharaonis						
Nitrosopumilus maritimus						
**CHLOROFLEXI— FIRMICUTES**									
Chloroflexus aggregans						
Chloroflexus aurantiacus						
Chloroflexus sp.						
Herpetosiphon aurantiacus						
Sphaerobacter thermophilus						
Bacillus methanolicus						
Bacillus smithii						
Bacillus sp.						
Caldalkalibacillus thermarum						
Geobacillus kaustophilus						
Geobacillus sp.						
Geobacillus thermodenitrificans						
Geobacillus thermoglucosidans						
Geobacillus thermoglucosidasius						
Sulfobacillus acidophilus						
Symbiobacterium thermophilum						
Thermaerobacter marianensis						
Thermaerobacter subterraneus						
Thermus scotoductus						
**ACTINOBACTERIA**									
Acidothermus cellulolyticus						
Actinobacillus minor						
Actinobacillus pleuropneumoniae						
Actinobacillus succinogenes						
Actinobacillus ureae						
Actinomyces coleocanis						
Actinomyces odontolyticus						
Actinomyces sp.						
Actinomyces urogenitalis						
Actinoplanes missouriensis						
Actinosynnema mirum						
Corynebacterium accolens						
Corynebacterium aurimucosum						
Corynebacterium diphtheriae						
Corynebacterium efficiens						
Corynebacterium pseudogenitalium						
Corynebacterium striatum						
Corynebacterium tuberculostearicum						
Micromonospora aurantiaca						
Micromonospora sp.						
Rubrobacter xylanophilus						
Thermobifida fusca						
**OTHER**									
Gemmatimonas aurantiaca						
Candidatus nitrospira						
Leptospira biflexa						
Turneriella parva						
Chthoniobacter flavus						
Methylacidiphilum fumariolicum						
Methylacidiphilum infernorum						

**Table A4:** **Primer hits for cd3af/R3cd and nirs1F/nirs6R with 0 and 2 mismatches to the *nirS* reference dataset**.

	**0 Mismatches**				**2 Mismatches**			
	**cd3af**	**R3cd**	**nirs1F**	**nirS6R**	**cd3af**	**R3cd**	**nirs1F**	**nirs6R**
**ALPHA-PROTEOBACTERIA**								
Dinoroseobacter shibae	x		x	x	x			x
Magnetospirillum gryphiswaldense	x				x	x	x	x
Magnetospirillum magneticum	x			x	x	x	x	x
Paracoccus denitrificans	x	x	x	x	x	x	x	x
Paracoccus pantotrophus	x	x	x	x	x	x	x	x
Polymorphum gilvum	x	x	x		x	x	x	x
Rhodobacter sp.		x		x	x	x	x	x
Roseobacter denitrificans			x	x	x		x	x
Roseobacter litoralis			x	x	x		x	x
Ruegeria pomeroyi			x	x	x		x	x
Stappia aggregate	x		x	x	x	x	x	x
**BETA-PROTEOBACTERIA**								
Achromobacter sp.	x	x	x		x	x	x	
Acidovorax delafieldii					x	x	x	
Acidovorax ebreus	x				x	x		x
Acidovorax sp.	x				x	x		x
Alicycliphilus denitrificans	x		x		x	x	x	x
Anaerolinea thermophile					x			x
Azoarcus sp.	x	x	x		x	x	x	x
Bordetella petrii		x			x	x	x	x
Brachymonas denitrificans			x		x	x	x	
Burkholderia cepacia	x	x	x	x	x	x	x	x
Candidatus accumulibacter				x	x	x	x	x
Comamonas denitrificans						x	x	
Cupriavidus metallidurans						x	x	x
Cupriavidus necator		x	x	x	x	x	x	x
Cupriavidus taiwanensis				x	x	x	x	x
Dechloromonas aromatica		x	x	x	x	x	x	x
Dechlorosoma suillum	x	x			x	x	x	x
Leptothrix cholodnii	x				x		x	x
Pseudogulbenkiania ferrooxidans				x	x	x	x	x
Pseudogulbenkiania sp.					x	x	x	x
Ralstonia eutropha		x	x	x	x	x	x	x
Rubrivivax benzoatilyticus	x		x		x	x	x	
Rubrivivax gelatinosus	x		x		x	x	x	
Sideroxydans lithotrophicus					x	x	x	x
Thauera sp.	x	x		x	x	x	x	x
Thiobacillus denitrificans	x	x	x	x	x	x	x	x
**GAMMA PROTEOBACTERIA**								
Beggiatoa sp.								
gamma proteobacterium			x		x		x	x
Hahella chejuensis	x				x		x	x
Kangiella koreensis								x
Marinobacter aquaeolei			x			x	x	x
Marinobacter hydrocarbonoclasticus	x				x	x	x	x
Pseudomonas aeruginosa	x	x	x	x	x	x	x	x
Pseudomonas brassicacearum	x				x		x	x
Pseudomonas chloritidismutans	x	x	x		x	x	x	
Pseudomonas fluorescens	x		x		x	x	x	x
Pseudomonas sp.	x	x	x	x	x	x	x	x
Pseudomonas stutzeri	x	x	x	x	x	x	x	x
nitratiruptor sp.								
**OTHER**								
Aromatoleum aromaticum	x	x			x	x	x	
Roseiflexus castenholzii				x	x			x
Oceanithermus profundus						x		x
Thermus scotoductus								x
Thermus thermophilus					x			x
Candidatus kuenenia								
Persephonella marina								
Sulfurihydrogenibium sp.								
Hydrogenivirga sp.						x		
Hydrogenobacter thermophilus						x		
Hydrogenobaculum sp.								
Rhodothermus marinus					x	x		
Candidatus methylomirabilis						x	x	x
uncultured bacterium	x	x			x			x
uncultured chloroflexi								x
